# P09 The use of ESR and pain scores to determine the duration of antibiotic therapy in patients with necrotizing otitis externa

**DOI:** 10.1093/jacamr/dlac004.008

**Published:** 2022-02-16

**Authors:** Yasir Sacranie, Wendy Smith

**Affiliations:** Kettering General Hospital NHS Trust, Kettering, Northamptonshire, NN16 8UZ; Kettering General Hospital NHS Trust, Kettering, Northamptonshire, NN16 8UZ

## Abstract

**Background:**

Diagnosis of necrotizing otitis externa is often delayed despite patients having severe pain disproportionate to the clinical findings as well as cranial nerve palsies. The incidence is increasing in elderly, diabetic or immunocompromised patients and can be fatal without ‘appropriate’ antibiotic therapy.^1,2^

**Objectives:**

CT, MRI, and technetium 99 bone scans are often performed but poorly correlate to the clinical response to empirical treatment.^3^ This novel study evaluated the use of erythrocyte sedimentation rate (ESR) and pain scores as measures of response to determine the dose and duration of antibiotic therapy in patients diagnosed with NOE over a 4 year period from November 2015.

**Methods:**

Patients diagnosed with NOE were evaluated using pain scores (0–10/10 where 0 = no pain and 10 = worst pain) and ESR at presentation and then at subsequent outpatient visits. Patients received 6 weeks of IV antibiotics (most receiving piperacillin/tazobactam 13.5 g daily via a 24 h infusion pump administered through a ‘mid-line’ as an outpatient when they were clinically well to go home) followed by oral ciprofloxacin. Initially ciprofloxacin was prescribed at 750 to 500 mg twice daily and the dose and duration of treatment was personalized according to pain scores and ESR. No ethical approval was required since this was an observational study of standard management of this condition at the Trust.

**Results:**

Twelve patients (10 males; 2 females) aged 49 to 91 years (median 74.5 years) were treated. Five had diabetes mellitus and three had been treated for a malignancy. The time of first symptoms to diagnosis ranged from 3 weeks to 32 months (median 3 months). Six had developed facial nerve palsies; three of whom had additional cranial nerve involvement. All had swabs positive for *Pseudomonas aeruginosa* which in four patients was resistant to gentamicin and in two of these patients also to ciprofloxacin. At presentation pain score was 10/10 in all but three patients (5/10 in one patient and 8/10 in two patients) and ESR values ranged from 31 to 82 (median 66). Both values fell within 1–2 weeks of commencing IV antibiotics and after 6 weeks pain scores were 0/10 in eight patients, 3/10 in the others. ESR at this stage fell to 10–33 (median 15). One patient developed diarrhoea and stopped ciprofloxacin after only 2 weeks remaining well at 6 months follow-up. Eight patients required 3 to 12 months ciprofloxacin. Four patients required restarting or increasing ciprofloxacin dose to control rising pain scores which reflected an increase in their ESR values but in one of these patients having grown *Pseudomonas* resistant to ciprofloxacin, two further 6 week courses of piperacillin/tazobactam were required. Three patients currently remain on ciprofloxacin at the lowest dose to keep their pain scores and ESR under control

**Conclusions:**

Pain scores with ESR values are invaluable in determining the activity of NOE thereby personalizing the antibiotic dose and duration in patients with this life-threatening infection.

